# Difference in root K^+^ retention ability and reduced sensitivity of K^+^-permeable channels to reactive oxygen species confer differential salt tolerance in three *Brassica* species

**DOI:** 10.1093/jxb/erw236

**Published:** 2016-06-23

**Authors:** Koushik Chakraborty, Jayakumar Bose, Lana Shabala, Sergey Shabala

**Affiliations:** ^1^Department of Plant Physiology, ICAR-Directorate of Groundnut Research, Junagadh, Gujarat-362 001, India; ^2^School of Land and Food and Tasmanian Institute for Agriculture, University of Tasmania, Hobart, Private Bag 94, Tas 7001, Australia

**Keywords:** H^+^-ATPase, ion homeostasis, membrane potential, potassium retention, ROS detoxification, sodium exclusion, tissue tolerance.

## Abstract

*Brassica* species are known to possess significant inter and intraspecies variability in salinity stress tolerance, but the cell-specific mechanisms conferring this difference remain elusive. In this work, the role and relative contribution of several key plasma membrane transporters to salinity stress tolerance were evaluated in three *Brassica* species (*B. napus*, *B. juncea*, and *B. oleracea*) using a range of electrophysiological assays. Initial root growth assay and viability staining revealed that *B. napus* was most tolerant amongst the three species, followed by *B. juncea* and *B. oleracea*. At the mechanistic level, this difference was conferred by at least three complementary physiological mechanisms: (i) higher Na^+^ extrusion ability from roots resulting from increased expression and activity of plasma membrane SOS1-like Na^+^/H^+^ exchangers; (ii) better root K^+^ retention ability resulting from stress-inducible activation of H^+^-ATPase and ability to maintain more negative membrane potential under saline conditions; and (iii) reduced sensitivity of *B. napus* root K^+^-permeable channels to reactive oxygen species (ROS). The last two mechanisms played the dominant role and conferred most of the differential salt sensitivity between species. *Brassica napus* plants were also more efficient in preventing the stress-induced increase in *GORK* transcript levels and up-regulation of expression of *AKT1*, *HAK5*, and *HKT1* transporter genes. Taken together, our data provide the mechanistic explanation for differential salt stress sensitivity amongst these species and shed light on transcriptional and post-translational regulation of key ion transport systems involved in the maintenance of the root plasma membrane potential and cytosolic K/Na ratio as a key attribute for salt tolerance in *Brassica* species.

## Highlight

This work provides the mechanistic explanation for differential salt stress sensitivity amongst *Brassica* species and links it with regulation of root plasma membrane potential and the cytosolic K/Na ratio

## Introduction

In today’s context of global climate change, salinization of arable land is a major threat to the agricultural production system. Although improving salt tolerance in major cultivated crops is of paramount importance to global food security in the 21st century, the process is significantly handicapped by the physiological and genetic complexity of the salinity tolerance trait and, as such, requires understanding of the orchestrated regulation of major subtraits at the tissue-specific level (Zhu, 2001).

Oilseeds and vegetables are two very important components of human food and are dietary necessities. The family *Brassicaceae* includes a number of cultivated crop species that have a considerable degree of variation in their salt tolerance (Purty *et al.*, 2008; Kumar *et al.*, 2009; Chakraborty *et al.*, 2016*a*). A considerable inter- and intraspecific variation in the overall growth, electrolyte leakage, proline accumulation, and maintenance of the K^+^/Na^+^ ratio was observed for different *Brassica* species (Ashraf and McNeilly, 2004; Purty *et al.*, 2008; Chakraborty *et al.*, 2012). At the whole-plant level, the mechanism of salt tolerance varied considerably among three *Brassica* species (*B. napus*, *B. juncea*, and *B. oleracea*) tested with 150mM NaCl stress (Chakraborty *et al.*, 2016*b*). It was shown that the overall superior salinity stress tolerance in *B. napus* was achieved by the higher osmo-tolerance matched by the moderate tissue tolerance and superior K^+^ retention ability in the leaf mesophyll. At the same time, while showing relatively modest overall tolerance, *B. oleracea* was far more superior in terms of shoot tissue tolerance. However, to the best of our knowledge, no specific details on the molecular/cellular mechanisms conferring this intra- and interspecific variability in salinity tolerance are available in the literature, and no significant quantitative trait locus (QTL) related to salinity tolerance has been identified in *Brassica* to date (Nayidu *et al.*, 2013).

For the majority of glycophytes, salinity tolerance is achieved through more than one strategy operating either simultaneously or in isolation, depending upon the duration and intensity of the stress (Munns, 2002). Such strategies include improved osmotic adjustment, exclusion of Na^+^ from uptake, intracellular Na^+^ sequestration, K^+^ retention in the cytosol, control of xylem ion loading, and oxidative stress tolerance (Ashraf *et al.*, 2008; Adem *et al.*, 2014; Bose *et al.*, 2014*a*). Which of these make the greatest contribution to salinity stress tolerance in *Brassica*?

Many glycophytes including cultivated crop species employ Na^+^ exclusion and/or partitioning strategies to achieve salt tolerance. Induced expression of the plasma membrane Na^+^/H^+^ antiporter (encoded by the *SOS1* gene in Arabidopsis) results in improved salt tolerance in several species (Zhu, 2001). In contrast, the Arabidopsis *salt overly sensitive1* (*sos1*) mutant, not having the capacity to exclude Na^+^ from its uptake, was found to accumulate 5-fold more Na^+^ in the shoot tissue and showed a highly sensitive phenotype (Nublat *et al.*, 2001). Unlike SOS1 which pumps cytosolic Na^+^ into the apoplastic space, the tonoplast-based NHX1 Na^+^/H^+^ antiporter removes cytotoxic Na^+^ by pumping it into the vacuole for sequestration (Fan *et al.*, 2015). At the same time, no significant difference in unidirectional (channel-mediated) Na^+^ uptake was reported between genotypes contrasting in salinity stress tolerance in various crop species [e.g. wheat (Davenport *et al.*, 2005) and barley (Chen *et al.*, 2007)]. Is this also the case for *Brassica*?

Another important aspect of achieving tissue tolerance is through maintenance of K^+^ homeostasis (Anschutz *et al.*, 2014; Shabala *et al.*, 2016*a*). By screening nearly 70 barley varieties contrasting in salinity stress tolerance, Chen *et al.* (2007) showed that >60% of genetic variability in salinity stress tolerance in barley was related to the roots’ ability to retain K^+^ in the mature root zone upon acute NaCl treatment. Similar reports were later published for wheat (Cuin *et al.*, 2008), lucerne (Smethurst *et al.*, 2008), and poplar (Sun *et al.*, 2009), and strong evidence for the inheritance of this trait was provided (Chen *et al.*, 2008; Cuin *et al.*, 2011). At the same time, NaCl-induced K^+^ efflux from wheat roots was shown to be an order of magnitude smaller compared with barley, for the same experimental conditions (for comparison, see Chen *et al.*, 2007; Cuin *et al.*, 2009). Does K^+^ retention in roots play a major role in salinity stress tolerance in *Brassica*? If the answer is yes, how is this K^+^ retention achieved? Does this occur at the transcriptional or post-translational level? Several pathways were reported to mediate NaCl-induced K^+^ efflux from plant tissues. These are highly tissue specific and include (Wu *et al.*, 2015; Shabala *et al.*, 2016*a*): (i) depolarization-activated outward-rectifying K^+^-selective channels; (ii) weakly voltage-dependent non-selective cation channels (NSCCs); and (iii) reactive oxygen species (ROS)-activated K^+^-permeable channels. Which of these pathways (if any) operates in *Brassica* roots?

Previous reports also suggested that cytosolic Ca^2+^ signalling is an essential component of plant adaptation to saline conditions (Dodd *et al.*, 2010). At least several concurrent signalling loops have to be considered. One of them is related to the salt overly sensitive (SOS) pathway. The activity of SOS1 is regulated by the CBL–CIPK complex that is achieved by phosphorylating its C-terminus (Quintero *et al.*, 2011). To enable this activation, a salt stress-induced Ca^2+^ signal is first detected by SOS3 (CBL4), a myristoylated calcium-binding protein; SOS3 is then activated by recruiting SOS2 (CIPK24), a serine/threonine protein kinase (reviewed in Shabala *et al.*, 2015). Elevated apoplastic Ca^2+^ levels are also essential to NSCC-mediated Na^+^ uptake (Demidchik and Tester, 2002) and prevent K^+^ leakage from the cell (Shabala *et al.*, 2006). Finally, salinity stress results in a substantial build-up of the cytotoxic hydroxyl radical and induces K^+^ efflux and Ca^2+^ influx in both root and shoot tissues (Demidchik *et al.*, 2003, 2010), with NADPH oxidase being one of the major sources of such ROS production and accumulation in the apoplast (Bose *et al.*, 2014*b*; Demidchik, 2015; Shabala *et al.*, 2015). Also, both constitutive and ROS-induced ion fluxes could vary significantly between different parts of the root (i.e. the mature and elongation zone) (Demidchik *et al.*, 2007). In this context, it was shown that the Ca^2+^ transport protein Annexin 1 in *Arabidopsis thaliana* (AtANN1) mediates root responses to extracellular H_2_O_2_ (Laohavisit *et al.*, 2012), and so are cyclic nucleotide-gated Ca^2+^-permeable channels (Ordonez *et al.*, 2014). What role do Ca^2+^ transport systems play in *Brassica* root responses to salinity?

The present study attempted to address the above questions by conducting a detailed electrophysiological investigation of mechanisms underlying interspecific variability in salinity stress tolerance in *Brassica*, at the tissue level. The non-invasive micro-electrode ion flux estimation (MIFE) technique was employed to study the kinetics of stress-induced net fluxes of Na^+^, K^+^, Ca^2+^, and H^+^ from various root zones and then link these changes with changes in the transcriptional profile of several key candidate genes. The molecular identity of the transporters involved was further confirmed in a series of pharmacological experiments.

## Materials and methods

### Plant material and experimental condition

The *Brassica* seeds of three different species, namely *B. napus*, *B. juncea*, and *B. oleracea*, were obtained from commercial suppliers (Zepson Seeds, Richmond and Hollander Imports, Hobart, Australia). Seeds were surface sterilized with 1% (v/v) HClO for 10min, thoroughly rinsed with distilled water, and then grown using the paper roll method (Pandolﬁ *et al.*, 2010) in non-buffered basic salt medium (BSM) solution (0.5mM KCl+0.2mM NaCl+0.1mM CaCl_2_, pH 5.7) in the dark for 4–5 d at room temperature (24±1°C). Plants were used for electrophysiological measurements when their root length was between 50mm and 70mm.

For long-term salinity treatment experiments [root growth assay, viability staining, membrane potential (MP), steady-state Na^+^ flux measurement, and gene expression studies], the seeds of all three *Brassica* species were surfaced sterilized and placed in paper rolls, and allowed to germinate and grow for 3 d in dark as described above. Then the uniformly grown seedlings were transferred to a 1.5 litre plastic container of aerated BSM added with the appropriate strength of NaCl as per the treatment requirement. The seedlings were suspended on a plastic grid so that their roots were completely immersed in the BSM. Plants were then grown under 16/8h light/dark cycle at 24 °C air temperature with an irradiance of 150 µmol m^−2^ s^−1^ for another 2–4 d.

### Root growth assay

Root length was measured from control and 150mM NaCl-treated plants, which were subjected to at least 48h of salt treatment. The reduction in the root length of salt-treated seedlings was expressed as a percentage of the control by comparing them with plants of the same age grown in BSM without salt.

### Viability staining

For viability staining, the *Brassica* seeds were grown in BSM without (control) and with NaCl added (treatment) as described above and the roots were collected after 2 d of 150mM NaCl treatment. The viability of the *Brassica* roots was assessed by using the fluorescein diacetate (FDA)–propidium iodide (PI) double staining method as described in Koyama *et al.* (1995). FDA (Cat. No. F7378; Sigma, St. Louis, MO, USA) is a non-polar ester permeable through the intact plasma membrane, and shows green colour under a fluorescent microscope in viable cells after hydrolysis by the internal esterases, while PI (Cat. No. P4864; Sigma-Aldrich) is impermeable to the intact plasma membrane and enters only cells with a damaged plasma membrane with large pores (i.e. dead or dying cells), which is indicated by red colour under a fluorescent light upon PI–nuclear DNA conjugate formation.

Control and 150mM NaCl-treated seedlings were stained in the darkness with freshly prepared FDA solution (5 µg ml^–1^) for 3min, followed by PI solution (3 µg ml^–1^) for 10min. The double-stained roots were observed under a florescence microscope (Leica MZ12; Leica Microsystems, Wetzlar, Germany) illuminated by an ultra-high-pressure mercury lamp (Leica HBO Hg 100W; Leica Microsystems) and fitted with a Leica I3-wavelength filter cube (Leica Microsystems). The excitation and emission wavelengths were 488/505–530nm and 543/585nm for FDA and PI, respectively. Photographs were taken with a Leica (DFC295; Leica Microsystems) camera fitted on the microscope using image acquisition and processing software LAS V3.8 (Leica Microsystems). During image acquisition, all the automatic exposure features of the LAS V3.8 were disabled, and exposure time (0.62s), gain (2.3×), saturation (0.35), and gamma (2.37) were set to constant values for all the measurements. To determine the extent of cell death, the red and green channels of the fluorescent images were separated and the fluorescent intensity of the respective images was quantified using Fiji (ImageJ) software.

### Ion flux measurements

Net ﬂuxes of H^+^, K^+^, Ca^2+^, and Na^+^ were measured by a non-invasive ion flux measurement technique using vibrating ion-selective microelectrodes (the MIFE technique; University of Tasmania) as described previously (Cuin *et al.*, 2011; Wu *et al.*, 2015). Briefly, microelectrodes were prepared from borosilicate glass capillaries by pulling capillaries followed by drying and silanization with tributylchlorosilane. The electrode tips were broken to achieve external tip diameters of 2–3 µm before they were backfilled with the corresponding back-filling solutions, followed by front-filling with appropriate ion-selective cocktails (see Supplementary Table S1 at *JXB* online).

Electrodes were mounted on a 3D-micromanipulator (MMT-5, Narishige) and calibrated in an appropriate set of standards encompassing measured ranges of particular ions using a three-point calibration. For MIFE measurements, the plant sample with intact roots (already adapted to BSM for 40min) was immobilized in a measuring chamber, mounted on a microscope stage, and electrode tips positioned 40 µm away from the root surface. Net fluxes were measured from the elongation (800–1000 µm from the root tip) and mature (20mm from the root tip) root zones. The electrochemical potential difference between the two positions was recorded for each electrode by the MIFE CHART software (Shabala *et al.*, 1997) and converted to ion concentration difference using the calibrated Nernst slope of the electrode. Net ion fluxes were calculated using the MIFEFLUX software for cylindrical diffusion geometry (Newman, 2001). All treatments [either 150mM NaCl for salinity stress; or 0.3mM CuCl_2_+1mM Na-ascorbate (referred to as Cu/A) for oxidative stress] were applied after recording steady fluxes of the respective ions for at least 5min. For the steady-state Na^+^ flux measurements, both the control and roots treated with 150mM NaCl for 48h were immobilized as described above and net fluxes were recorded for at least 5min from the mature and elongation zones.

### Pharmacology

In pharmacological experiments, plant roots were pre-treated for 30–60min prior to application of NaCl or Cu/A stresses with one of the following: 1mM sodium orthovanadate (vanadate; a potent blocker of the H^+^-ATPase pump; Sigma, Cat. S6508); 1mM amiloride (a potent blocker of Na^+^/H^+^ antiport; Sigma Cat. A4562); or 1 µM Eosin Yellow (EY; a known blocker of the Ca^2+^-ATPase pump; Sigma Cat. 119830).

### Membrane potential measurement

Both control and salt-treated seedlings were immobilized in a measuring chamber as described earlier (Bose *et al.*, 2014*a*) and were kept for pre-conditioning in BSM (with/without NaCl) for 50–60min. A conventional microelectrode (GC 150F-10, Harvard Apparatus Ltd, Kent, UK) with a tip diameter of ~0.5 µm was filled with 0.5M KCl and connected to a MIFE electrometer (Shabala *et al.*, 1997) via an Ag–AgCl half-cell. The mounted electrode was then impaled in the external cortex cells of intact roots using a manually operated hydraulic micromanipulator (MHW-4-1; Narishige, Tokyo, Japan). The steady-state MP measurements were conducted from at least six individual roots (from both the mature and elongation zones as described above) with no more than four impalements per root undertaken. Each measurement was averaged for at least a 30s interval (Bose *et al.*, 2014*a*).

### Gene expression study

For gene expression studies, *Brassica* seedlings grown as described earlier were subjected to 150mM NaCl stress for a period for 1h and 48h in order to simulate the short- and long-term salt stress condition, which finally correlated with ion flux measurement and other root growth studies. Total RNAs from root tissues of control and salt-treated plants were isolated using the RNASure Plant Kit (Genetix Brand, Cat NP-84905) according to the manufacturer’s protocol, with minor modifications. Briefly, root tissues (~100mg) were homogenized in liquid nitrogen to a fine powder and suspended in lysis buffer, before they were subjected to on-column DNase digestion to eliminate any genomic DNA contamination. Finally, the RNA pellet was re-suspended in 20 µl of RNase-free water and stored at −20 °C for further use. The integrity of the RNA was confirmed in the gel, and the absorbance of the isolated RNA was recorded using a Nanodrop Spectrophotometer (ND 1000). Approximately 2 µg of total RNA was used for cDNA synthesis using a First strand cDNA synthesis kit (Thermo Scientific), and the synthesized cDNA was confirmed by PCR using all cDNA samples (~100ng) as template with an 18S rRNA primer. To test gene-specific primers (see Supplementary Table S2 for sequence details), the above experiment was carried out for each set of primers and a single amplicon PCR product was confirmed in agarose gel.

Changes in expression of the above genes (transcript levels) were studied by real-time quantitative PCR (StepOnePlus™ Real-Time PCR System from Applied Biosystems using a QuantiFast SYBR Green PCR kit, Qiagen,USA). The reaction mixture included ~100ng of cDNA, 0.16 µM of primers, and 12.5 µl of QuantiFast SYBR Green PCR mix. The reaction volume was maintained at 20 µl by sterile nuclease-free water. Reactions were run under the following conditions: 95 °C for 5min for one cycle; 95 °C for 10s and 60 °C for 30s for 40 cycles. At the end of the PCR cycles, the products were put through a melt curve analysis to determine the specificity of amplification. The fold changes in transcript in salt-treated roots were compared with those of control plants in terms of fold change, analysed by the comparative 2^–ΔΔCt^ method (Schmittgen and Livak, 2008). The *Br-18s rRNA* gene was used as the internal control to normalize the PCRs (Sarkar *et al.*, 2014).

## Results

### Differential salt sensitivity was observed in three *Brassica* species under both short- and long-term salinity stress

**Fig. 1.**
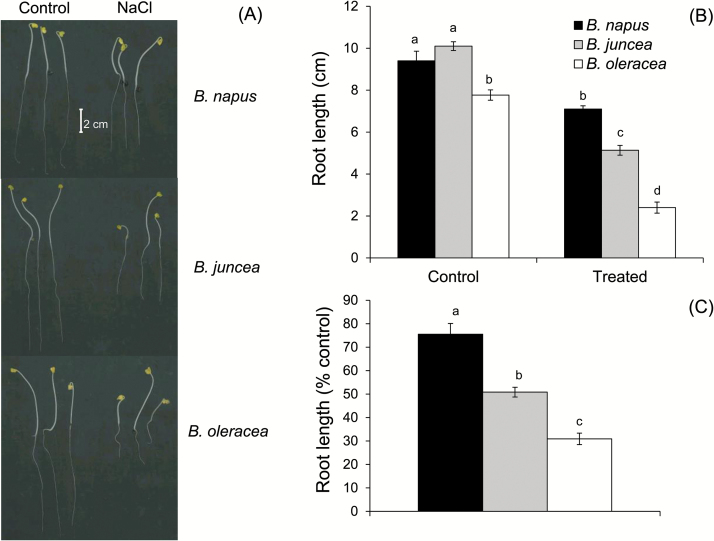
Effect of salinity stress on root growth of three *Brassica* species (5-day-old seedling) grown in non-buffered basic salt medium (BSM) solution (0.5mM KCl+0.2mM NaCl+0.1mM CaCl_2_, pH 5.7) and exposed to 150mM NaCl stress for 72h. Values are the mean ±SE (*n*=5 individual plants).

The three *Brassica* species (*B. napus*, *B. juncea*, and *B. oleracea*) do vary significantly in terms of tolerance to salinity stress when studied in long-term experiments under glasshouse or field conditions (Ashraf and McNeilly, 2004; Chakraborty *et al.*, 2016*b*). This differential salt sensitivity is phenotypically detected even under a much shorter duration of stress. Significant differences in the root growth rate were found in 5-day-old seedlings grown under control and saline (150mM NaCl) conditions (Fig. 1). The visible difference in the root growth (Fig. 1A) of salt-treated plants suggested the order of salt tolerance as *B. napus*> *B. juncea*>*B. oleracea*, at the seedling stage. Under control conditions, the root growth varied between 8cm and 10cm in 5-day-old seedlings of the three *Brassica* species tested. Treatment with 150mM NaCl restricted root growth to a mere ~30% and ~50% in the case of *B. oleracea* and *B. juncea*, respectively, while there was nearly 75% retention of root length in *B. napus* compared with control plants (Figs 1B, C).

**Fig. 2.**
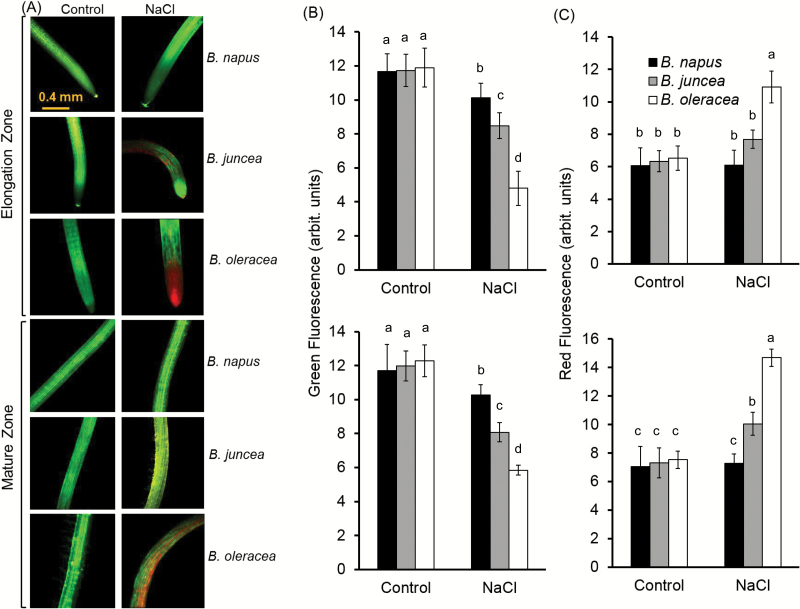
Viability staining of the elongation and mature root zones of three *Brassica* species exposed to 150mM NaCl stress for 48h. (A) One (of five) typical image is shown for each treatment/species. (B, C) Intensity of the green (B) and red (C) fluorescent signal. Values are the mean ±SE (*n*=15–20).

The three *Brassica* species showed distinctive variability in the tissue-specific sensitivity to salt stress, as revealed by viability staining experiments (Fig. 2A). After 48h of 150mM NaCl treatment, the root tip of *B. napus* (most tolerant) was almost as healthy as non-treated root, while both *B. juncea* (>25% mortality) and *B. oleracea* (>50% mortality) showed a considerably higher level of mortality around the root tip (Fig. 2B, C). Unlike *B. napus* and *B. juncea*, *B. oleracea*, showed symptoms of dying from the root tip spreading towards the mature zone upon long-term salt exposure. Similar to the elongation zone, the mature zone of all the three species showed loss of viability to different extents, with *B. napus* being the least damaged (~10%), while *B. juncea* (~30%) and *B. oleracea* (~50%) exhibited comparatively much higher loss of viability (Fig. 2B, C).

### Mature and elongation zones behaved differently in terms of Na^+^ exclusion and sensitivity towards salt stress

**Fig. 3.**
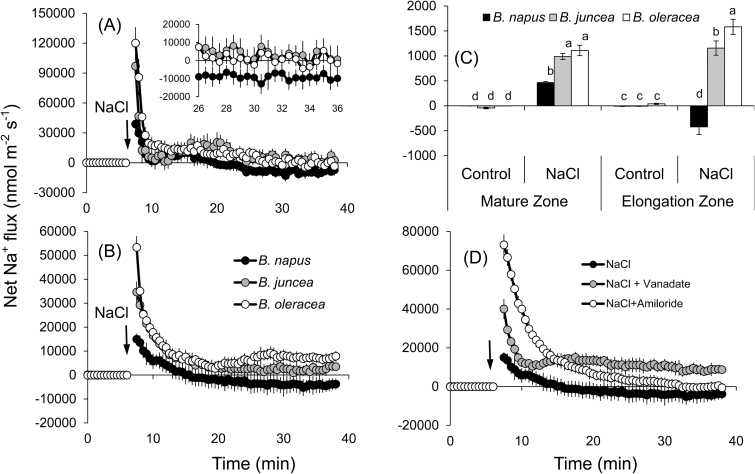
Transient net Na^+^ flux kinetics measured in three *Brassica* species from elongation (A) and mature (B) root zones in response to 150mM NaCl stress. The inset in (A) resolves a genotypic difference in the net Na^+^ efflux at the end of the transient recording. (C) Steady-state Na^+^ efflux from roots (both mature and elongation zones) of three *Brassica* species exposed to 150mM NaCl stress for 48h. (D) Transient net Na^+^ flux kinetics in response to 150mM NaCl treatment measured from the mature root zone of *B. napus* pre-treated for 1h in solutions containing a specific metabolic inhibitor or a channel blocker. Values are the mean ±SE (*n*=6–8). The sign convention for all MIFE measurements is ‘efflux negative’.

Addition of 150mM NaCl to the root medium resulted in a massive Na^+^ uptake in plant roots (Fig. 3), in both the elongation (Fig. 3A) and mature (Fig. 3B) zones, although with rather different magnitude (~2-fold higher in the root apex). This uptake gradually slowed down, and net Na^+^ efflux was measured from *B. napus* roots at ~15min after onset of 150mM NaCl stress in both zones. In the other two species, however, net Na^+^ flux significantly decreased (to nearly zero) but never turned into net efflux (Fig. 3A, B). Also different were the peak magnitudes of net Na^+^ uptake immediately after NaCl application. In the mature zone (the major bulk of the root), the most tolerant *B. napus* had a peak value of 15 000±2200 nmol m^−2^ s^−1^, while it was much higher in *B. juncea* (>2-fold) and *B. oleracea* (>3.5-fold). Also, *B. napus* was able to extrude Na^+^ in the mature zone (Fig. 3B), while both *B. juncea* and *B. oleracea* showed net Na^+^ uptake of ~3500 nmol m^−2^ s^−1^ and ~8000 nmol m^−2^ s^−1^, respectively, 30min after the salt treatment. The above findings reported for transient Na^+^ fluxes were further confirmed in experiments involving long-term salinity treatments. Root exposure to 150mM NaCl for 48h showed that *B. napus* had 2-fold less steady-state Na^+^ uptake in the mature zone compared with the two other *Brassica* species (Fig. 3C) and was the only species that showed a statistically significant net Na^+^ efflux of ~425 nmol m^−2^ s^−1^ in the elongation zone (Fig. 3C), while the other two species showed only net Na^+^ uptake (Fig. 3C).

A series of pharmacological experiments was conducted to reveal the identity of the above strong Na^+^ exclusion mechanism in *B. napus*. Pre-treatment of roots with sodium orthovanadate (a known blocker of H^+^-ATPase) and amiloride (an inhibitor of the Na^+^/H^+^ plasma membrane exchanger; Cuin *et al.*, 2011) for 1h completely changed the pattern of Na^+^ fluxes in *B. napus* roots (Fig. 3D). Both chemicals shifted net Na^+^ fluxes towards more positive values by increasing the peak magnitude of net Na^+^ uptake by >2.5-fold and ~5-fold for vanadate and amiloride treatment, respectively. Also, no net Na^+^ efflux was measured in either treatment at the end of the transient recordings (Fig. 3D). While control plants showed a net Na^+^ efflux of −3800±2700 nmol m^−2^ s^−1^ after 30min of NaCl treatment, amiloride-treated roots had a net Na^+^ uptake of 1500±1250 nmol m^−2^ s^−1^. These results strongly suggest that the Na^+^ exclusion mechanism in *B. napus* is mediated by the plasma membrane-localized Na^+^/H^+^ antiporter fuelled by H^+^-ATPase.

### K^+^ flux kinetics in mature and elongation zones upon salinity treatment

**Fig. 4.**
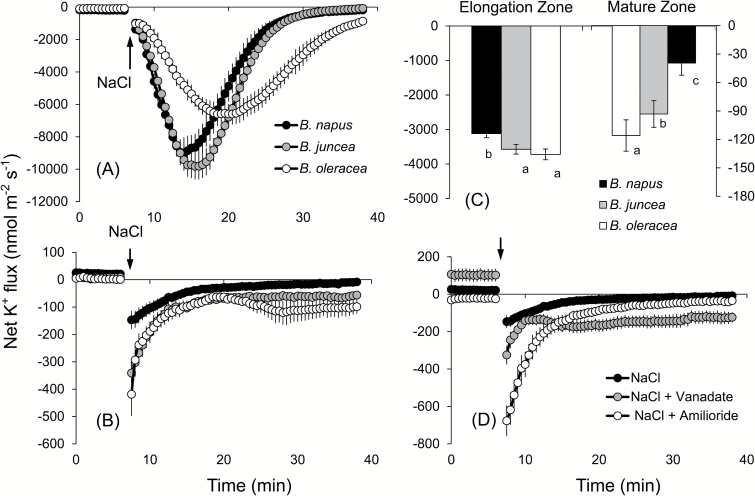
Transient net K^+^ flux kinetics measured in three *Brassica* species from the elongation (A) and mature (B) root zones in response to 150mM NaCl stress. (C) Average K^+^ flux from roots over the 30min period after Na^+^ addition to three *Brassica* species (both mature and elongation zones). (D) Transient net K^+^ flux kinetics in response to 150mM NaCl treatment measured from the mature root zone of *B. napus* pre-treated for 1h in solutions containing specific metabolic inhibitors. Values are the mean ±SE (*n*=6–8).

The strong efflux of K^+^ was observed upon addition of NaCl to the bath medium in both the elongation and mature zones of all the three species, although the pattern differed significantly between the two root zones (Fig. 4A, B). In the elongation zone, the peak K^+^ efflux was highest in *B. junce*a followed by *B. napus* and *B. oleracea*. The salt-induced K^+^ leakage was slowly restored to the pre-stress values in *B. napus* and *B. juncea* 25min after the stress application, while K^+^ efflux in *B. oleracea* persisted throughout the duration of the flux measurements (up to 40min in Fig. 4A; indicated as negative flux values). We also observed a delay in the timing of peak K^+^ efflux which occurred 5±1min (*B. napus*), 7±0.5min (*B. juncea*), and 14±2min (*B. oleracea*) after application of the stress (Fig. 4A). The overall amount of K^+^ leaked over the 30min interval since the stress (calculated as the area under the curve in the respective graphs) was the smallest in the most tolerant *B. napus* (Fig. 4C, left panel; significant at *P*<0.05). In the mature zone, the highest K^+^ efflux (peak value) was observed immediately after the imposition of a salt stress. This flux was slowly restored to pre-stress values in *B. napus* (Fig. 4B) while it remained highly negative (net K^+^ efflux) in the other two varieties (significant at *P*<0.05). The magnitude of the peak K^+^ efflux also varied between the three species, with minimum values observed in *B. napus* followed by *B. juncea* and *B. oleracea.* Similar to the elongation zone, the overall salt-induced K^+^ leakage was the highest in the most salt-sensitive species *B. oleracea* and the least leakage was observed in the most salt-tolerant *B. napus* (Fig. 4C, right panel). Pre-treatment of *B. napus* (the most salt tolerant among the varieties tested) with vanadate resulted in a higher peak K^+^ efflux and the loss of the ability to restore K^+^ values to the pre-stress level (Fig. 4D), suggesting involvement of H^+^-ATPase. Pre-treatment of roots with 1mM amiloride led to a >4-fold increase in the peak K^+^ efflux and remained higher (more negative) than the respective value without the inhibitor 30min after the treatment (−80±15 nmol m^−2^ s^−1^ and 0±8 nmol m^−2^ s^−1^, respectively). Taken together, the observed results suggest involvement of the voltage-gated K^+^ channels in K^+^ retention in *Brassica* roots under salt stress.

### *Brassica napus* spends more energy through active H^+^ pumping for maintaining the membrane potential under salinity stress

**Fig. 5.**
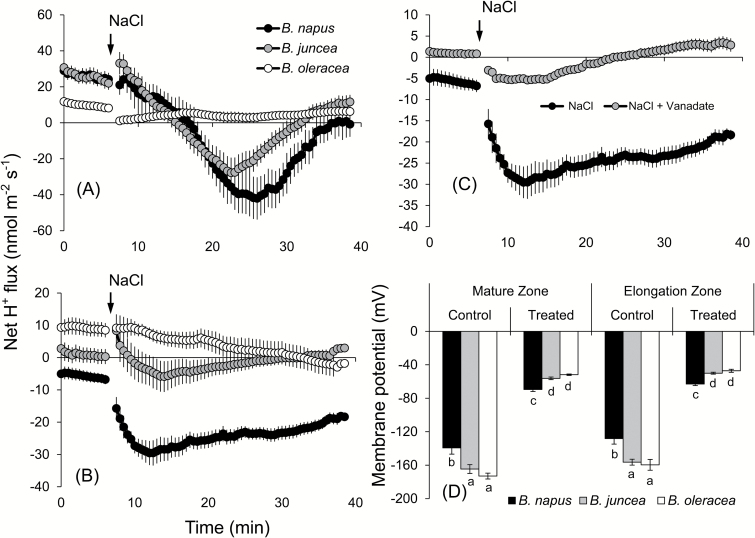
Transient net H^+^ flux kinetics measured in three *Brassica* species from elongation (A) and mature (B) root zones in response to 150mM NaCl stress. (C) Transient net H^+^ flux kinetics in response to 150mM NaCl treatment measured from the mature root zone of *B. napus* pre-treated for 1h with 1mM sodium orthovanadate. Values are the mean ±SE (*n*=6–8). (D) Membrane potential (mV) of epidermal root cells of three *Brassica* species measured from roots exposed to 150mM NaCl stress for 48h. Mean ±SE (*n*=20–25).

The onset of salinity stress led to a significant shift in H^+^ fluxes towards more negative values in all species and both root zones assessed. The magnitude of this shift, however, showed a clear species and tissue dependence (Fig. 5). In the elongation zone, all three species had net initial uptake (zero to 5min; Fig. 5A). Addition of NaCl led to a shift towards H^+^ efflux. This shift was strongest in *B. napus* followed by *B. juncea* and then by *B. oleracea* (Fig. 5A). A similar pattern was also observed in the mature zone (Fig. 5B), although here the steady-state initial H^+^ flux values were more negative in all species (net efflux of −6±0.9 nmol m^−2^ s^−1^ in *B. napus* and 0.2±1.1 nmol m^−2^ s^−1^ in *B. juncea*, compared with ~22±1.8 nmol m^−2^ s^−1^ in both species in the elongation zone. The peak H^+^ flux values in the mature zone were −30±4, −6±4, and −2.5±2 nmol m^−2^ s^−1^ for *B. napus*, *B. juncea*, and *B. oleracea*, respectively (Fig. 5B), and −42±12, −28±8, and 0±2 nmol m^−2^ s^−1^ in the elongation zone (Fig. 5A).

The NaCl activation of H^+^ pumping was largely prevented by vanadate which reduced H^+^ efflux peak values from −30±4 nmol m^−2^ s^−1^ to −5±1 nmol m^−2^ s^−1^ upon vanadate pre-treatment (demonstrated as an example for *B. napus*; Fig. 5C). Also affected was the steady-state initial H^+^ flux (negative in the control; slightly positive after vanadate pre-treatment). Taken together, these results point towards the involvement of H^+^-ATPase. Given the fact that the plasma membrane H^+^-ATPase is a critical player in determining the MP of plant cells (Palmgren and Niessen, 2011), MP values were tested in three varieties under control and salt treatment (48h NaCl) conditions. The largest decrease in MP values was observed in *B. oleracea* in both the mature (from −170±4 mV in control to −50±0.8 mV after salt treatment) and elongation (from −160±6.5 to −47±1.8 mV) zones, respectively (Fig. 5D). *Brassica. napus* not only showed the least reduction in MP under long-term salt stress, but also was able to maintain the highest MP under 150mM salt treatment in both the mature (−70±2.5 mV) and elongation (−63±1.9 mV) zones.

### ROS-induced K^+^ leakage is related to the active efflux of Ca^2+^ through Ca^2+^-ATPase

**Fig. 6.**
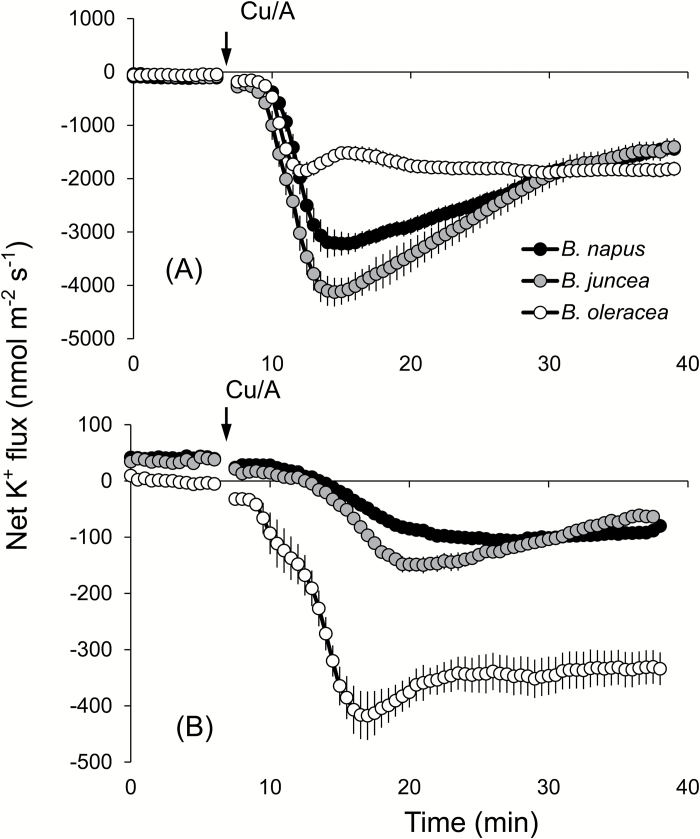
Transient net K^+^ flux kinetics measured in three *Brassica* species from the elongation (A) and mature (B) root zones in response to oxidative (1mM Cu/A) stress. Values are the mean ±SE (*n*=4–6).

Production of ROS under salinity stress is inevitable, and thus a plant’s ability to withstand ROS stress is often associated with the overall salt tolerance (Bose *et al.*, 2014*b*). To assess the sensitivity of the three *Brassica* species to oxidative stress, the seedlings were subjected to hydroxyl radical generation by Cu/A treatment (Demidchik *et al.* 2003, 2010). Addition of Cu/A to the BSM solution resulted in a profuse K^+^ efflux, in both the mature and elongation zones (Fig. 6). The magnitude of this efflux differed significantly between the two zones, with almost a 10-fold higher efflux observed from the elongation zone. Here, the peak values of K^+^ efflux occurred 7–8min after the treatment (Fig. 6A). In the mature zone, the extent of Cu/A activation of the K^+^ efflux was inversely correlated with the overall salinity stress tolerance (*B. oleracea*>*B. juncea*>*B. napus*) (Fig. 6B).

**Fig. 7.**
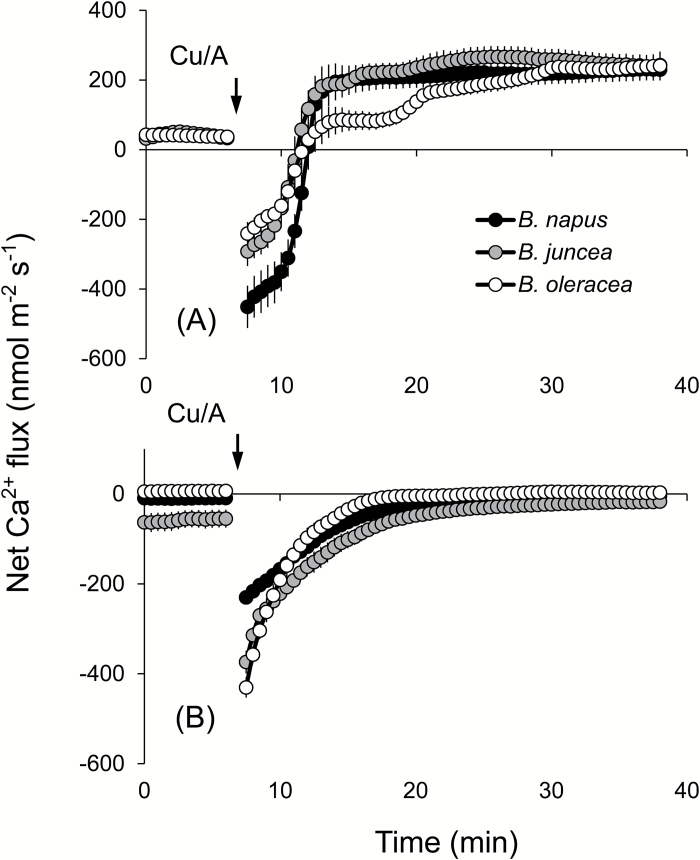
Transient net Ca^2+^ flux kinetics measured in three *Brassica* species from the elongation (A) and mature (B) root zones in response to oxidative (1mM Cu/A) stress. Values are the mean ±SE (*n*=4–6).

**Fig. 8.**
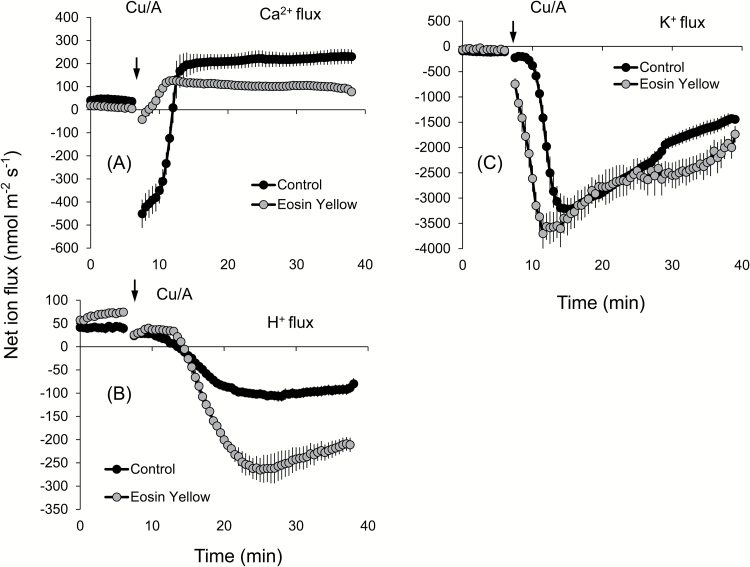
Transient net Ca^2+^ (A), H^+^ (B), and K^+^ (C) flux kinetics measured in response to oxidative (1mM Cu/A) stress from the elongation root zone of *B. napus* pre-treated for 1h in solutions containing 1 µM Eosin Yellow, a known blocker of the Ca^2+^-ATPase pump. Values are the mean ±SE (*n*=4–6).

ROS stress is known to affect Ca^2+^ homeostasis in plants (Demidchik *et al.*, 2010; Zepeda-Jazo *et al.*, 2011), with possible implications for salinity stress signalling. Therefore, we measured the kinetics of net Ca^2+^ fluxes in response to Cu/A treatment. Of all ROS produced under saline conditions, the hydroxyl radical is the most aggressive and cannot be controlled by means of enzymatic antioxidants (for a review, see Bose *et al.*, 2014*b*). In addition, due to the signalling role of H_2_O_2_ in plant adaptive responses (Gilroy *et al.*, 2014; Niu and Liao, 2016; Shabala *et al.*, 2016*b*), it is often rather difficult to separate ‘detrimental’ and ‘beneficial’ effects of H_2_O_2_ on the activity of ion transporters. Taken together, these two facts determined our use of the hydroxyl radical as a ROS agent in this work. Application of the Cu/A hydroxyl radical-generating mix to *Brassica* roots stimulated net Ca^2+^ uptake in the elongation zone (Fig. 7A) but resulted in a gradually decaying net Ca^2+^ efflux from the mature zone (Fig. 7B). No clear differences between species have emerged. Interestingly, immediately upon Cu/A application, a brief transient Ca^2+^ efflux was also measured in all three *Brassica* species for several minutes, before turning into net Ca^2+^ uptake (Fig. 7A). Due to the ~100-fold higher Ca^2+^ concentration gradient across the plasma membrane, the passive Ca^2+^ efflux is thermodynamically impossible and could either originate from the cell wall (as a result of the Donnan exchange; Kinraide, 1998; Shabala and Newman, 2000) or be mediated by some active plasma membrane Ca^2+^ efflux system. To differentiate between these two possible sources, we pre-treated roots with Eosin Yellow (EY), a known inhibitor of Ca^2+^-ATPase (Romani *et al.*, 2004; Beffagna *et al.*, 2005). Pre-treatment of *B. napus* roots with EY completely blocked the initial Ca^2+^ efflux, suggesting involvement of Ca-ATPase in active Ca^2+^ pumping out from the cells (Fig. 7C). EY was also found to shorten the timing of the response to Cu/A, with the peak K^+^ efflux in the presence of EY occurring 3min prior to the peak values without EY in the elongation zone (Fig. 8A), and exacerbated the extent of Cu/A-induced activation of net K^+^ efflux from the mature zone (Fig. 8B).

### Selective induction of key ion transporters/pumps in *B. napus* explains its higher salinity tolerance among *Brassica* species

**Fig. 9.**
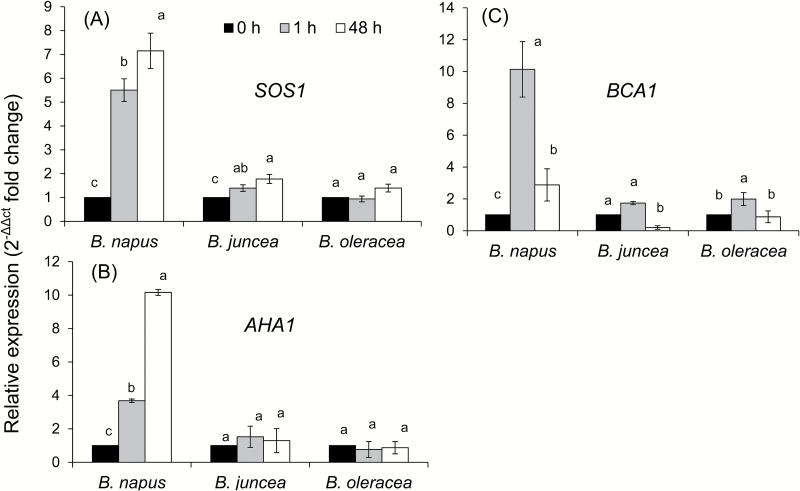
Quantitative real-time PCR analysis of the time-dependent expression pattern of *SOS1* (A), *AHA1* (B), and *BCA1* (C) genes in roots of three *Brassica* species grown in non-buffered basic salt medium (BSM) solution (0.5mM KCl+0.2mM NaCl+0.1mM CaCl_2_, pH 5.7) and exposed to 150mM NaCl stress for 0, 1, and 48h. The total RNA was extracted collectively from at least five individual plants for each treatment combination. Values are the mean ±SE (*n*=6 independent real-time PCRs performed). The relative fold-change values are given as the mean ±SE in each case.

Quantitative real-time PCR analyses were carried out to assess the relative abundance of the transcript level in control (0h) and after short-term (1h) and long-term (48h) salt stress in the three *Brassica* species. As postulated from the ion flux data and pharmacological tests, we found a significant induction of the plasma membrane Na^+^/H^+^-antiporter (*SOS1*) transcript in *B. napus*, after both 1h and 48h of 150mM salt treatment. The relative induction of *SOS1* transcript was ~5.5- and ~7-fold after 1h and 48h from the onset of salt stress, respectively (Fig. 9A), while it was induced only marginally in *B. juncea* (1.4- and 1.8-fold induction 1h and 48h after salt treatment, respectively). In the case of *B. oleracea*, the transcript level was not increased 1h after the treatment, while it was increased slightly (~1.4-fold) 48h afterwards. Upon exposure to salt stress, the transcript level of the plasma membrane *H* ^*+*^ *-ATPase* (*AHA1*) was also induced sharply only in *B. napus* (3.7- and 10-fold induction 1h and 48h after onset of the stress, respectively) (Fig. 9B). Neither *B. juncea* nor *B. oleracea* showed any significant (*P*<0.05) up-regulation of the *AHA1* transcript. Unlike *SOS1* and *AHA1* genes, the expression of the *Ca-ATPase* gene did not show a continuous increasing pattern with the increase of the salt treatment period (Fig. 9C). The highest induction of the *Ca-ATPase* transcript was observed in *B. napus*, 1h after the salt treatment (~10-fold), which decreased after 48h salt treatment (compared with 1h treatment) while still remaining ~3-fold higher than the control value. The minimal induction of *Ca-ATPase* transcript was observed in *B. juncea*, that was not statistically significant.

**Fig. 10.**
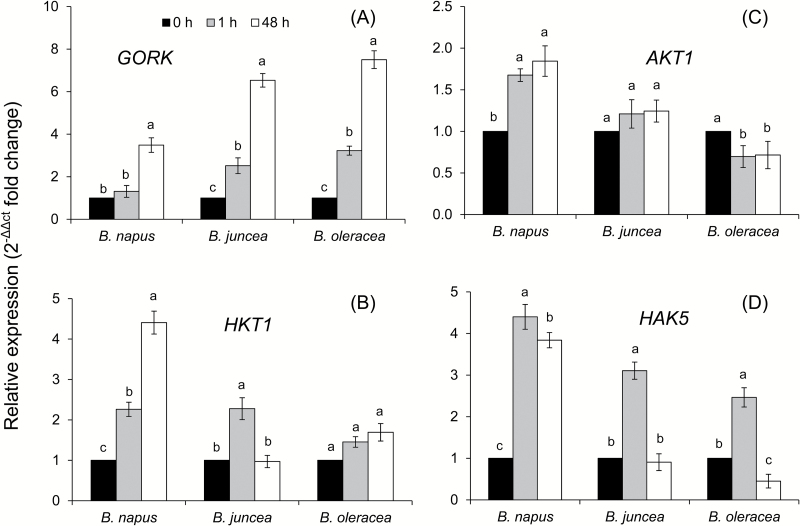
Quantitative real-time PCR analysis of the time-dependent expression pattern of *GORK* (A), *HKT1* (B), *AKT1* (C), and *HAK5* (D) genes in roots of three *Brassica* species grown in non-buffered basic salt medium (BSM) solution (0.5mM KCl+0.2mM NaCl+0.1mM CaCl_2_, pH 5.7) and exposed to 150mM NaCl stress for 0, 1, and 48h. The total RNA was extracted collectively from at least five individual plants for each treatment combination. Values are the mean ± SE (*n*=6–9 independent real-time PCRs performed). The relative fold-change values are given as the mean ±SE in each case.

The expression patterns of other key cellular ion transporters mediating intracellular K^+^ and Na^+^ homeostasis were also investigated (Fig. 10). Our first candidate was the GORK channel that mediates salt stress-induced K^+^ efflux from the cell in many plant species (Shabala and Cuin, 2008; Shabala and Pottosin, 2014). Salinity stress induced *GORK* transcript levels in all three *Brassica* species, in a time-dependent manner (Fig. 10A). After 1h of salt stress, there was no induction of *GORK* observed in *B. napus*, while there was a >2.5-fold induction in both *B. juncea* and *B. oleracea* (Fig. 10A). Although 48h of NaCl stress led to significant induction in *GORK* transcript abundance even in *B. napus*, the magnitude of induction was ~2-fold lower compared with the other two species. The quantitative PCR data on the relative transcript abundance of *HKT1*, *AKT1*, and *HAK5* showed a comparatively higher level of induction in *B. napus* for all three genes (Fig. 10B–D). In fact, the *AKT1* transcript did not show any significant change in either *B. juncea* or *B. oleracea* upon exposure to salt stress, while *HKT1* showed induction upon 1h of salt exposure in *B. juncea*, but no significant induction in *B. oleracea*. Strong up-regulation (although with differential magnitude) of the *HAK5* transcript was observed in all three *Brassica* species with short-term salt stress (1h), but it was either decreased to the initial level or even down-regulated with a longer duration (48h) of stress, except in *B. napus*, which was able to maintain ~3.8-fold up-regulation of the *HAK5* transcript level even after 48h of stress.

## Discussion

Similar to other cultivated glycophyte species, *Brassica* are also sensitive to salinity stress and possess a certain degree of variability in salinity tolerance at the species level (Ashraf *et al.*, 2001; Kumar *et al.*, 2009). Genetically, such variability in salt tolerance in *Brassica* is thought to be associated with the ploidy level of the species (Ashraf and McNeilly, 2004). In the present study, we found distinctive differences in salt tolerance of the three *Brassica* species studied. The root growth assay suggested the order of salt tolerance as *B. napus*>*B. juncea*>*B. oleracea* (Fig. 1); this was further confirmed by the viability staining data (Fig. 2). While these findings are consistent with some previous observations (Ashraf *et al.*, 2001; Purty *et al.*, 2008), no explanation for this intraspecies differential sensitivity to salt was given until now, at the mechanistic level. Here we provide strong evidence that higher salt tolerance in *B. napus* is conferred by at least three complementary physiological mechanisms: (i) higher Na^+^ extrusion ability from roots resulting from increased expression and activity of plasma membrane SOS1-like Na^+^/H^+^ exchangers; (ii) better root K^+^ retention ability resulting from stress-inducible activation of H^+^-ATPase and the ability to maintain a more negative MP under saline conditions; and (iii) reduced sensitivity of *B. napus* root K^+^-permeable channels to ROS. Of these, the two latter mechanisms played the dominant role in conferring salinity stress tolerance in *Brassica*. The supporting arguments are given below.

### SOS1-mediated Na^+^ extrusion may be essential for higher salt tolerance in *B. napus*

A massive influx of Na^+^ was observed in all three *Brassica* species immediately after imposition of salinity stress. Given the strong cytotoxicity of Na^+^ (Munns and Gilliham, 2015), plant performance under saline conditions will be critically dependent on the ability of *Brassica* species to remove excessive Na^+^ from the cytosol, either to the external media or by sequestering it into the vacuole. In contrast to mammalian systems, higher plants do not harbour Na^+^ ATPase pumps (Maathuis, 2014), and the above exclusion is believed to be mediated by Na^+^/H^+^ exchangers [either SOS1 at the plasma membrane (Shi *et al*., 2000) or NHX at the tonoplast membrane (Blumwald *et al.*, 2000)]. Here we provide strong evidence that superior salinity stress tolerance in *B. napus* is conferred by higher SOS1 activity. Indeed, *B. napus* was the only species showing active Na^+^ exclusion in both the mature and elongation root zones (Fig. 3A, B). This Na^+^ efflux was found to be sensitive to both vanadate (a known inhibitor of H^+^ -ATPase that provides a driving force for Na^+^/H^+^ antiporter operation) and amiloride [a known blocker of Na^+^/H^+^ exchangers in both mammalian (Grinstein *et al.* 1988) and plant (Cuin *et al.*, 2011) systems] (Fig. 3D), strongly suggesting the involvement of SOS1. This is further confirmed by measuring stress-induced changes in SOS1 transcript levels (Fig. 9) that were several fold greater in *B. napus* compared with the two other *Brassica* species. Also strongly induced was AHA1 expression (a 10-fold increase in transcript level), conferring the possibility to fuel the SOS1 operation. Thus, it is plausible to suggest that *B. napus* possesses a very efficient salt-sensing system that is able to translate changes in the rhizosphere conditions into both transcriptional (Fig. 9) and functional (Fig. 3) changes in the SOS1 activity. Specific details of the mechanisms behind thhe observed activation remain obscure. Zhu (2003) has argued that SOS1 protein has a long tail that resides in the cytoplasm and, by analogy with bacterial and yeast systems, may potentially operate as an Na^+^ sensor *per se*. However, no direct experimental support for this hypothesis has been provided so far, and it was argued (Shabala *et al.*, 2015) that SOS1 activity is regulated by a SOS3/SOS2 complex, which activates its C-terminus from the cytosolic site and thus relies on changes in cytosolic free Ca^2+^ (not external Na^+^) concentrations. Given the fact that changes in SOS1 activity strongly correlated with changes in net K^+^ and H^+^ fluxes, involvement of the H^+^-ATPase/GORK tandem system as a potential Na^+^ sensor (Shabala *et al.*, 2015) may be plausible. This issue is discussed in more detail in the following sections. It should be also noted that net Na^+^ efflux was not observed in either the elongation or the mature zone of the other two *Brassica* species (Fig. 3C), and thus cannot be responsible for the reported higher tolerance of *B. juncea* as compared with *B. oleracea* (Figs 1, 2). Thus, it is suggested that Na^+^ exclusion from uptake plays an important but not a crucial role as a determinant of genetic variability in salinity stress tolerance in *Brassica*.

### Maintenance of K^+^ homeostasis is critical for salt tolerance in *B. napus*

Maintenance of K^+^ homeostasis is essential for enzyme activities, ionic and pH homeostasis, and charge balance (Dreyer and Uozumi, 2011), and cytosolic K^+^ is considered to be the common denominator of plant adaptive responses to a broad range of environmental stresses (Shabala and Pottosin, 2014; Shabala *et al.*, 2016*a*). Changes in the cytosolic K^+^ are also suggested to be an important signal that may determine the cell’s fate and also switch its operation from the metabolic to defence mode (Demidchik *et al.*, 2014). Both X-ray crystallographic and ion-selective microelectrode-based measurements of the cytosolic K^+^ level were shown to be decreased considerably under salinity stress (Flowers and Hajibagheri, 2001; Cuin *et al.*, 2003), and a strong correlation between the root’s K^+^ retention ability and plant salinity stress tolerance was reported for several species including wheat (Cuin *et al.*, 2008, 2012), barley (Chen *et al.*, 2005, 2007; Wu *et al.*, 2015), poplar (Sun *et al.*, 2009), and lucerne (Smethurst *et al.*, 2008). Here we extend this list and show that the above mechanism is also applicable to explain the intraspecific variability in salinity tolerance in *Brassica*. Indeed, the average total K^+^ leak was found to be in the order *B. napus*<*B. juncea*<*B. oleracea*, which was in an agreement with the overall ability of these species to tolerate external salt stress (Fig. 4). A strong correlation (see Supplementary Table S3) between K^+^ retention and net Na^+^ uptake observed in the *Brassica* species (especially in the mature root zone); sensitivity of NaCl-induced K^+^ efflux to vanadate and amiloride (Fig. 4D); and a positive correlation between root K^+^ retention ability and plasma membrane potential under saline conditions (Fig. 5D) collectively point towards involvement of GORK channels as a major pathway for the salt stress-induced K^+^ efflux from *Brassica* roots. GORK (outward-rectifying potassium selective) channels are activated by membrane depolarization (Very *et al.*, 2014), and their gating is strongly dependent upon the extracellular K^+^ concentration (Anschutz *et al.*, 2014). GORK channels are ubiquitously expressed in Arabidopsis tissues and represent an essential element in volume control of various cell types such as guard cells, pollen tube, or root hairs (Becker *et al.*, 2003; Hosy *et al.*, 2003). They are also known to be ROS sensitive and determine cell fate by mediating induction of programmed cell death under saline conditions (Demidchik *et al.*, 2010). These findings reported for Arabidopsis are in a very good agreement with our data reporting much smaller activation of K^+^ efflux by the hydroxyl radical-generating Cu/A mix in *B. napus* roots (Fig. 6B) compared with the two other species. Thus, it may be concluded that the intraspecific variability in salinity stress tolerance among *Brassica* species is critically dependent on the functional expression and/or regulation of GORK channels under saline conditions.

Interestingly, *GORK* transcript levels were up-regulated by 4- to 7-fold, in a clear time-dependent manner (Fig 10A). This is counterintuitive and contradicts the above notion of the essentiality of K^+^ retention in salinity stress tolerance. The most likely explanation is the comment made above that plants may use K^+^ efflux as a switch from metabolic to defence mode (Demidchik *et al.*, 2014), and increased expression of GORK channels may assist plants in achieving this goal. As such a switch has a caveat of losing too much K^+^, a very fine balance between the amount of K^+^ lost in this signalling process and the amount of K^+^ retained in the root for turgor and/or metabolic purposes is needed. *Brassica napus* plants seem to be very efficient in maintaining such a balance. Not only is the reported increase in *GORK* transcript 2-fold lower in *B. napus* compared with the other two species, but *B. napus* plants showed much higher transcript levels of both low (*AKT1*) and high (*HAK5*) affinity transport systems involved in K^+^ acquisition (hence, restoring cytosolic K^+^ levels once the signalling is over). Also 2 - to 3-fold higher were the *HKT1* transcript levels in *B. napus* 48h after stress onset (Fig. 10B). Traditionally, the role of HKT1 is attributed to retrieval of Na^+^ from the xylem (Munns and Tester, 2008). This suggest a better Na^+^ retention in the root of *B. napus* and a potential for its use for osmotic (turgor maintenance) purposes, to compensate for K^+^ lost.

### Maintenance of a higher membrane potential under salinity stress is achieved by an active H^+^ pumping by *B. napus*

An active H^+^ pumping is the key to maintaining the negative MP required for optimal plant growth (Sze *et al.*, 1999). Under saline conditions, NaCl induces activation of both plasma membrane and vacuolar H^+^-ATPases that are required to restore the otherwise depolarized MP in both halophytes and glycophytes (Yang *et al.*, 2007; Bose *et al.*, 2015). Here we show that the differential sensitivity of *Brassica* species to salinity stress is determined by the extent of NaCl-induced activation of H^+^-ATPase, at both the transcriptional (Fig. 9) and post-translational (Fig. 5) level.

A significant correlation between membrane depolarization and K^+^ leakage from the root tissue was established in earlier reports (Chen *et al.*, 2007; Bose *et al.*, 2014*a*) and attributed to the depolarization-activated outward-rectifying K^+^ channels (Shabala and Pottosin, 2014). In agreement with this, a dramatic K^+^ efflux was observed in all *Brassica* species tested (Fig. 4) that correlated with the extent of MP depolarization (Fig. 5). The NaCl-induced H^+^ efflux was almost instantaneous (Fig. 5B) and strongly blocked by vanadate, a known inhibitor of H^+^-ATPase (Fig. 5C). The causal link between the cytosolic K^+^ concentration and H^+^-ATPase activity was suggested by Buch-Pedersen *et al.* (2006) who showed that the plant plasma membrane H^+^-ATPase is regulated by potassium bound to the proton pump at a site involving Asp617 in the cytoplasmic phosphorylation domain, suggesting a role for K^+^ as an intrinsic uncoupler. Thus, it was suggested that GORK channels may be located in close proximity to H^+^-ATPase, forming a ‘microdomain’ in the lipid raft (Shabala *et al.*, 2015), and that a reduction in the cytosolic K^+^ concentration in the H^+^-ATPase microdomain may affect operation of the H^+^ pump as described above. This GORK/H^+^-ATPase ‘tandem’ may then operate as a salt sensor and explain the differential sensitivity between *Brassica* species.

### Differential ROS sensitivity in salt stress signalling in *Brassica*

Plants growing in a saline environment are facing cytotoxic ROS produced as a secondary effect of salinity stress. Demidchik *et al.* (2010) reported a 2.5- to 3-fold increase in hydroxyl radicals in Arabidopsis roots exposed to 100mM NaCl stress. Also the amount of H_2_O_2_ accumulated in the roots showed a significant increase under salt stress (Xie *et al.*, 2011). This increased load of ROS under saline conditions was shown to activate GORK channels, which conduct large outwardly rectifying K^+^ currents and thus increase K^+^ leakage from the tissue (Demidchik *et al.*, 2010). In full agreement with this, we report a high degree of K^+^ leakage in the mature zone of all three *Brassica* as soon as they are exposed to oxidative stress (Fig. 6B). The most salt-sensitive species, *B. oleracea*, not only showed the highest K^+^ efflux but the rate of induction of K^+^ leakage was also much faster in this species (judged by the timing of peak K^+^ efflux). Overall, K^+^ retention capacity in the mature root zone under oxidative stress (imposed by the hydroxyl radical-generating Cu/A mix; Demidchik *et al.*, 2003) was *B. napus*>*B. juncea*>*B. oleracea* as the order of tolerance, which is similar to the respective order for root growth and root viability under salt stress (Figs 1, 2). Interestingly, the pattern of K^+^ efflux was not similar in the elongation zone (Fig. 6A). In fact, it was almost the other way around, with *B. juncea* and *B. napus* showing higher K^+^ efflux than *B. oleracea*. Again, it may be tempting to suggest, therefore, that ROS-induced K^+^ leak from the root apex may perform a ‘positive’ (signalling) role, either switching cell metabolism from the metabolic into defence mode of operation (Demidchik *et al.*, 2014), thus enabling a rapid plant adaptation to altered conditions in the rhizosphere, or initiating a long-distance (root to shoot) signalling cascade (Shabala *et al.*, 2016*b*).

Oxidative stress is known also to affect Ca^2+^ homeostasis (Demidchik *et al.*, 2003) through a large number of Ca^2+^ transport systems (Demidchik *et al.*, 2010; Zepeda-Jazo *et al.*, 2011). Accordingly, ROS-induced net Ca^2+^ fluxes were also measured and assessed in this work. A rapid initial efflux of Ca^2+^ was observed from both the mature and elongation zones of *Brassica* roots immediately upon exposure to the oxidative stress (Fig. 7A, B). In the elongation zone, the highest Ca^2+^ efflux was observed in *B. napus*, which was converted into steady uptake of Ca^2+^ 7–12min after the stress application. For the other two species, the trend was also similar, with a slight difference between the periods of efflux in these species. As passive (channel-mediated) Ca^2+^ efflux is thermodynamically not possible, the reported data suggest a rapid activation by ROS of some active Ca^2+^ efflux systems, which is more pronounced in the salt-tolerant *B. napus* species. Cellular membranes harbour a large number of Ca^2+^ efflux systems (Ca^2+^-ATPase family) and Ca^2+^ exchangers (CAX family) (McAinsh and Pittman, 2009; Bose *et al.*, 2011), and activation of plasma membrane-based Ca^2+^-ATPase pumps by hydroxyl radicals was demonstrated in pharmacological and patch–clamp experiments in pea root cells (Zepeda-Jazo *et al.*, 2011). Accordingly, we have hypothesized that a similar scenario may be applicable to *Brassica* roots. Pharmacological tests revealed that the ROS-induced Ca^2+^ efflux was sensitive to EY, a known blocker of Ca^2+^-ATPase. The activation of Ca^2+^-ATPase also happened to be the highest in the elongation zone of *B. napus* and the lowest in the mature zone of *B. napus*, suggesting tissue-specific Ca^2+^ signalling in this species. The observed efflux is transient and is gradually transformed into net Ca^2+^ uptake that most probably results from the hydroxyl radical-induced activation of Ca^2+^-permeable channels in root plasma membranes, as reported in direct patch–clamp experiments on other species (Demidchik *et al.*, 2003; Foreman *et al.*, 2003; Demidchik and Maathuis, 2007; Zepeda-Jazo *et al.*, 2011). Taken together, a concurrent (or very closely timed) activation of both Ca^2+^ efflux and Ca^2+^ influx systems may shape the cytosolic Ca^2+^ signature, conferring specificity of the stress signal (Knight and Knight, 2001; Demidchik *et al.*, 2002; Ludwig *et al.*, 2004; Demidchik and Maathuis, 2007; Dodd *et al.*, 2010). Consistent with this suggestion were changes in BCA1 transcript levels that peaked at 1h and declined afterwards (once the signalling was over). The reported induction in BCA1 transcript was significantly higher in *B. napus* (Fig. 9C), consistent with the reported strongest net Ca^2+^ efflux from its roots Fig. 7A).

## Conclusions

To summarize, the superior salinity stress tolerance of *B. napus* was conferred by its better ability to exclude Na^+^ and retain K^+,^ thus providing an optimal cytosolic K/Na ratio. This ability was determined by the efficient transcriptional and post-translational regulation of key transport systems, namely the plasma membrane H^+^-ATPase pump, the SOS1 Na^+^/H^+^ exchanger, P-type Ca^2+^-ATPase, and low- and high-affinity K^+^ uptake systems. Specific details of their regulation and salt stress perception and signalling pathways remain to be revealed in follow-up studies.

## Supplementary data

Supplementary data are available at *JXB* online.

Figure S1. Propidium iodide-stained *B. oleracea* root apex showing dead cells after 48h of 150mM NaCl stress.

Table S1. List of liquid ionophores and respective back-filling solution used for micro-electrode preparation.

Table S2. Nucleotide sequences of different primers used in the study along with amplicon length.

Table S3. Correlation matrix (two-tailed Pearson’s correlation) for different physiological and ion uptake parameters with transcript abundance of key transporters/proteins

Supplementary Data

## References

[CIT0001] Adem GD Roy SJ Zhou M Bowman JP Shabala S 2014 Evaluating contribution of ionic, osmotic and oxidative stress components towards salinity tolerance in barley. BMC Plant Biology 14, 113. 24774965 10.1186/1471-2229-14-113 PMC4021550

[CIT0002] Anschütz U Becker D Shabala S 2014 Going beyond nutrition: regulation of potassium homoeostasis as a common denominator of plant adaptive responses to environment. Journal of Plant Physiology 171, 670–687. 24635902 10.1016/j.jplph.2014.01.009

[CIT0003] Ashraf M Athar HR Harris PJC Kwon TR 2008 Some prospective strategies for improving crop salt tolerance. Advances in Agronomy 97, 45–110.

[CIT0004] Ashraf M McNeilly T 2004 Salinity tolerance in *Brassica* oilseeds. Critical Reviews in Plant Science 23, 157–174.

[CIT0005] Ashraf M Nazir N McNeilly T 2001 Comparative salt tolerance of amphidiploid and diploid *Brassica* species. Plant Science 160, 683–689. 11448743 10.1016/s0168-9452(00)00449-0

[CIT0006] Becker D Hoth S Ache P Wenkel S Roelfsema MR Meyerhoff O Hartung W Hedrich R 2003 Regulation of the ABA-sensitive Arabidopsis potassium channel gene GORK in response to water stress. FEBS Letters 554, 119–126. 14596925 10.1016/s0014-5793(03)01118-9

[CIT0007] Beffagna N Buffoli B Busi C 2005 Modulation of reactive oxygen species production during osmotic stress in Arabidopsis thaliana cultured cells: involvement of the plasma membrane Ca^2+^-ATPase and H^+^-ATPase. Plant and Cell Physiology 46, 1326–1339. 15937326 10.1093/pcp/pci142

[CIT0008] Blumwald E Aharon GS Apse MP 2000 Sodium transport in plant cells. Biochimica et Biophysica Acta 1465, 140–151. 10748251 10.1016/s0005-2736(00)00135-8

[CIT0009] Bose J Pottosin II Shabala SS Palmgren MG Shabala S 2011 Calcium efflux systems in stress signaling and adaptation in plants. Frontiers in Plant Science 2, 85. 22639615 10.3389/fpls.2011.00085 PMC3355617

[CIT0010] Bose J Rodrigo-Moreno A Lai D Xie Y Shen W Shabala S 2015 Rapid regulation of the plasma membrane H^+^-ATPase activity is essential to salinity tolerance in two halophyte species, *Atriplex lentiformis* and *Chenopodium quinoa* . Annals of Botany 115, 481–494. 25471095 10.1093/aob/mcu219 PMC4332608

[CIT0011] Bose J Rodrigo-Moreno A Shabala S 2014 *b* ROS homeostasis in halophytes in the context of salinity stress tolerance. Journal of Experimental Botany 65, 1241–1257. 24368505 10.1093/jxb/ert430

[CIT0012] Bose J Shabala L Pottosin I Zeng F Velarde-Buendía A Massart A Poschenrieder C Hariadi Y Shabala S 2014 *a* Kinetics of xylem loading, membrane potential maintenance, and sensitivity of K^+^-permeable channels to reactive oxygen species: physiological traits that differentiate salinity tolerance between pea and barley. Plant, Cell and Environment 37, 589–600. 10.1111/pce.12180 23937055

[CIT0013] Buch-Pedersen MJ Rudashevskaya EL Berner TS Venema K Palmgren MG 2006 Potassium as an intrinsic uncoupler of the plasma membrane H^+^-ATPase. Journal of Biological Chemistry 281, 38285–38292. 17056603 10.1074/jbc.M604781200

[CIT0014] Chakraborty K Bose J Shabala L Eyles A Shabala S 2016 *b* Evaluating relative contribution of osmo- and tissue-tolerance mechanisms towards salinity stress tolerance in three Brassica species. Physiologia Plantarum. doi:10.1111/ppl.12447. 10.1111/ppl.12447 27062083

[CIT0015] Chakraborty K Sairam RK Bhaduri D 2016 *a* Effects of different levels of soil salinity on yield attributes, accumulation of nitrogen, and micronutrients in *Brassica* spp. Journal of Plant Nutrition 39, 1026–1037.

[CIT0016] Chakraborty K Sairam RK Bhattacharya RC 2012 Salinity induced expression of pyrrolline-5-carboxylate synthetase determine salinity tolerance in *Brassica* spp. Acta Physiologiae Plantarum 34, 1935–1941.

[CIT0017] Chen Z Newman I Zhou M Mendham N Zhang G Shabala S 2005 Screening plants for salt tolerance by measuring K^+^ flux: a case study for barley. Plant, Cell and Environment 28, 1230–1246.

[CIT0018] Chen Z Pottosin II Cuin TA 2007 Root plasma membrane transporters controlling K^+^/Na^+^ homeostasis in salt-stressed barley. Plant Physiology 145, 1714–1725. 17965172 10.1104/pp.107.110262 PMC2151677

[CIT0019] Chen Z Shabala S Mendham N Newman I Zhang G Zhou M 2008 Combining ability of salinity tolerance on the basis of NaCl-induced K flux from roots of barley. Crop Science 48, 1382–1388.

[CIT0020] Cuin TA Betts SA Chalmandrier R Shabala S 2008 A root’s ability to retain K^+^ correlates with salt tolerance in wheat. Journal of Experimental Botany 59, 2697–2706. 18495637 10.1093/jxb/ern128 PMC2486465

[CIT0021] Cuin TA Bose J Stefano G Jha D Tester M Mancuso S Shabala S 2011 Assessing the role of root plasma membrane and tonoplast Na^+^/H^+^ exchangers in salinity tolerance in wheat: in planta quantification methods. Plant, Cell and Environment 34, 947–961. 10.1111/j.1365-3040.2011.02296.x 21342209

[CIT0022] Cuin TA Miller AJ Laurie SA Leigh RA 2003 Potassium activities in cell compartments of salt-grown barley leaves. Journal of Experimental Botany 54, 657–661. 12554708 10.1093/jxb/erg072

[CIT0023] Cuin TA Tian Y Betts SA Chalmandrier R Shabala S 2009 Ionic relations and osmotic adjustment in durum and bread wheat under saline conditions. Functional Plant Biology 36, 1110–1119. 10.1071/FP09051 32688722

[CIT0024] Cuin TA Zhou M Parsons D Shabala S 2012 Genetic behaviour of physiological traits conferring cytosolic K^+^⁄ Na^+^ homeostasis in wheat. Plant Biology 14, 438–446. 22117736 10.1111/j.1438-8677.2011.00526.x

[CIT0025] Davenport R James RA Zakrisson-Plogander A Tester M Munns R 2005 Control of sodium transport in durum wheat. Plant Physiology 137, 807–818. 15734907 10.1104/pp.104.057307 PMC1065380

[CIT0026] Demidchik V 2015 Mechanisms of oxidative stress in plants: from classical chemistry to cell biology. Environmental and Experimental Botany 109, 212–228.

[CIT0027] Demidchik V Cuin TA Svistunenko D Smith SJ Miller AJ Shabala S Sokolik A Yurin V 2010 Arabidopsis root K^+^-efflux conductance activated by hydroxyl radicals: single-channel properties, genetic basis and involvement in stress-induced cell death. Journal of Cell Science 123, 1468–1479. 20375061 10.1242/jcs.064352

[CIT0028] Demidchik V Davenport RJ Tester M 2002 Nonselective cation channels in plants. Annual Review of Plant Biology 53, 67–107. 10.1146/annurev.arplant.53.091901.161540 12221989

[CIT0029] Demidchik V Maathuis FJM 2007 Physiological roles of nonselective cation channels in plants: from salt stress to signalling and development. New Phytologist 175, 387–404. 17635215 10.1111/j.1469-8137.2007.02128.x

[CIT0030] Demidchik V Shabala SN Coutts KB Tester MA Davies JM 2003 Free oxygen radicals regulate plasma membrane Ca^2+^- and K^+^-permeable channels in plant root cells. Journal of Cell Science 116, 81–88. 12456718 10.1242/jcs.00201

[CIT0031] Demidchik V Shabala SN Davies JM 2007 Spatial variation in H_2_O_2_ response of *Arabidopsis thaliana* root epidermal Ca^2+^ flux and plasma membrane Ca^2+^ channels. The Plant Journal 49, 377–386. 17181775 10.1111/j.1365-313X.2006.02971.x

[CIT0032] Demidchik V Straltsova D Medvedev SS Pozhvanov GA Sokolik A Yurin V 2014 Stress-induced electrolyte leakage: the role of K^+^-permeable channels and involvement in programmed cell death and metabolic adjustment. Journal of Experimental Botany 65, 1259–1270. 24520019 10.1093/jxb/eru004

[CIT0033] Demidchik V Tester M 2002 Sodium fluxes through nonselective cation channels in the plasma membrane of protoplasts from *Arabidopsis* roots. Plant Physiology 128, 379–387. 11842142 10.1104/pp.010524 PMC148901

[CIT0034] Dodd AN Kudla J Sanders D 2010 The language of calcium signaling. Annual Review of Plant Biology 61, 593–620. 10.1146/annurev-arplant-070109-104628 20192754

[CIT0035] Dreyer I Uozumi N 2011 Potassium channels in plant cells. FEBS Journal 278, 4293–4303. 21955642 10.1111/j.1742-4658.2011.08371.x

[CIT0036] Fan W Deng G Wang H Zhang H Zhang P 2015 Elevated compartmentalization of Na^+^ into vacuoles improves salt and cold stress tolerance in sweet potato (*Ipomoea batatas*). Physiologia Plantarum 154, 560–571. 25307930 10.1111/ppl.12301

[CIT0037] Flowers TJ Hajibagheri MA 2001 Salinity tolerance in *Hordeum vulgare*: ion concentrations in root cells of cultivars differing in salt tolerance. Plant and Soil 231, 1–9.

[CIT0038] Foreman J Demidchik V Bothwell JHF 2003 Reactive oxygen species produced by NADPH oxidase regulate plant cell growth. Nature 422, 442–446. 12660786 10.1038/nature01485

[CIT0039] Gilroy S Suzuki N Miller G Choi WG Toyota M Devireddy AR Mittler R 2014 A tidal wave of signals: calcium and ROS at the forefront of rapid systemic signaling. Trends in Plant Science 19, 623–630. 25088679 10.1016/j.tplants.2014.06.013

[CIT0040] Grinstein S Smith JD Onizuka R Cheung RK Gelfand EW Benedict S 1988 Activation of Na^+^/H^+^ exchange and the expression of cellular proto-oncogenes in mitogen- and phorbol ester-treated lymphocyte. Journal of Biological Chemistry 263, 8658–8665. 2837462

[CIT0041] Hosy E Vavasseur A Mouline K 2003 The Arabidopsis outward K+ channel GORK is involved in regulation of stomatal movements and plant transpiration. Proceedings of the National Academy of Sciences, USA 100, 5549–5554. 10.1073/pnas.0733970100 PMC154382 12671068

[CIT0042] Kinraide TB 1998 Three mechanisms for the calcium alleviation of mineral toxicities. Plant Physiology 118, 513–520. 9765536 10.1104/pp.118.2.513 PMC34826

[CIT0043] Knight H Knight MR 2001 Abiotic stress signalling pathways: specificity and cross-talk. Trends in Plant Science 6, 262–267. 11378468 10.1016/s1360-1385(01)01946-x

[CIT0044] Koyama H Toda T Yokota S Dawair Z Hara T 1995 Effects of aluminium and pH on root growth and cell viability in *Arabidopsis thaliana* strain Landsberg in hydroponic culture. Plant and Cell Physiology 36, 201–205.

[CIT0045] Kumar G Purty RS Sharma MP Singla-Pareek SL Pareek A 2009 Physiological responses among *Brassica* species under salinity stress show strong correlation with transcript abundance for SOS pathway-related genes. Journal of Plant Physiology 166, 507−520. 18799232 10.1016/j.jplph.2008.08.001

[CIT0046] Laohavisit A Shang Z Rubio L 2012 Arabidopsis annexin1 mediates the radical-activated plasma membrane Ca^2+^- and K^+^-permeable conductance in root cells. The Plant Cell 24, 1522–1533. 22523205 10.1105/tpc.112.097881 PMC3398561

[CIT0047] Ludwig AA Romeis T Jones JD 2004 CDPK-mediated signalling pathways: specificity and cross-talk. Journal of Experimental Botany 55, 181–188. 14623901 10.1093/jxb/erh008

[CIT0048] Maathuis FJ 2014 Sodium in plants: perception, signalling, and regulation of sodium fluxes. Journal of Experimental Botany 65, 849–858. 24151301 10.1093/jxb/ert326

[CIT0049] McAinsh MR Pittman JK 2009 Shaping the calcium signature. New Phytologist 181, 275–294. 19121028 10.1111/j.1469-8137.2008.02682.x

[CIT0050] Munns R 2002 Comparative physiology of salt and water stress. Plant, Cell and Environment 25, 239–250. 10.1046/j.0016-8025.2001.00808.x 11841667

[CIT0051] Munns R Gilliham M 2015 Salinity tolerance of crops—what is the cost? New Phytologist 208, 668–673. 26108441 10.1111/nph.13519

[CIT0052] Munns R Tester M 2008 Mechanisms of salinity tolerance. Annual Review of Plant Biology 59, 651–681. 10.1146/annurev.arplant.59.032607.092911 18444910

[CIT0053] Nayidu N Bollina V Kagale S 2013 Oilseed crop productivity under salt stress. In: Ahmad P Azouz MM Prasad MNV, eds. Ecophysiology and responses of plants under salt stress. Heidelberg: Springer, 249–265.

[CIT0054] Newman IA 2001 Ion transport in plants: measurement of fluxes using ion-selective microelectrodes to characterize transporter function. Plant, Cell and Environment 24, 1–14. 10.1046/j.1365-3040.2001.00661.x 11762438

[CIT0055] Niu LJ Liao WB 2016 Hydrogen peroxide signaling in plant development and abiotic responses: crosstalk with nitric oxide and calcium. Frontiers in Plant Science 7, 230. 26973673 10.3389/fpls.2016.00230 PMC4777889

[CIT0056] Nublat A Desplans J Casse F Berthomieu P 2001 Sas1, an Arabidopsis mutant over-accumulating sodium in the shoot, shows deficiency in the control of the root radial transport of sodium. The Plant Cell 13, 125–137. 11158534 10.1105/tpc.13.1.125 PMC102204

[CIT0057] Ordoñez NM Marondedze C Thomas L Pasqualini S Shabala L Shabala S Gehring C 2014 Cyclic mononucleotides modulate potassium and calcium flux responses to H_2_O_2_ in Arabidopsis roots. FEBS Letters 588, 1008–1015. 24530500 10.1016/j.febslet.2014.01.062

[CIT0058] Palmgren MG Nissen P 2011 P-Type ATPases. Annual Review of Biophysics 40, 243–266. 10.1146/annurev.biophys.093008.131331 21351879

[CIT0059] Pandolfi C Pottosin I Cuin T Mancuso S Shabala S 2010 Specificity of polyamine effects on NaCl-induced ion flux kinetics and salt stress amelioration in plants. Plant and Cell Physiology 51, 422–434. 20061303 10.1093/pcp/pcq007

[CIT0060] Purty RS Kumar G Singla-Pareek SL Pareek A 2008 Towards salinity tolerance in *Brassica*: an overview. Physiology and Molecular Biology of Plants 14, 39–49. 23572872 10.1007/s12298-008-0004-4 PMC3550665

[CIT0061] Quintero FJ Martinez-Atienza J Villalta I 2011 Activation of the plasma membrane Na/H antiporter Salt-Overly-Sensitive 1 (SOS1) by phosphorylation of an auto-inhibitory C-terminal domain. Proceedings of the National Academy of Sciences, USA 108, 2611–2616. 10.1073/pnas.1018921108 PMC3038701 21262798

[CIT0062] Romani G Bonza MC Filippini I Cerana M Beffagna N De Michelis MI 2004 Involvement of the plasma membrane Ca^2+^-ATPase in the short-term response of *Arabidopsis thaliana* cultured cells to oligogalacturonides. Plant Biology 6, 192–200. 15045671 10.1055/s-2004-817848

[CIT0063] Sarkar T Thankappan R Kumar A Mishra GP Dobaria JR 2014 Heterologous expression of the *AtDREB1A* gene in transgenic peanut-conferred tolerance to drought and salinity stresses. PLoS One 9, e110507. 25545786 10.1371/journal.pone.0110507 PMC4278701

[CIT0064] Schmittgen TD Livak KJ 2008 Analyzing real-time PCR data by the comparative CT method. Nature Protocols 3, 1101–1108. 18546601 10.1038/nprot.2008.73

[CIT0065] Shabala S Bose J Fuglsang AT Pottosin I 2016 *a* On a quest for stress tolerance genes: membrane transporters in sensing and adapting to hostile soils. Journal of Experimental Botany 67, 1015–1031. 26507891 10.1093/jxb/erv465

[CIT0066] Shabala S Cuin TA 2008 Potassium transport and plant salt tolerance. Physiologia Plantarum 133, 651–669. 18724408 10.1111/j.1399-3054.2007.01008.x

[CIT0067] Shabala S Demidchik V Shabala L Cuin TA Smith SJ Miller AJ Davies JM Newman IA 2006 Extracellular Ca^2+^ ameliorates NaCl-induced K^+^ loss from Arabidopsis root and leaf cells by controlling plasma membrane K^+^-permeable channels. Plant Physiology 141, 1653–1665. 16798942 10.1104/pp.106.082388 PMC1533937

[CIT0068] Shabala S Newman I 2000 Salinity effects on the activity of plasma membrane H^+^ and Ca^2+^ transporters in bean leaf mesophyll: masking role of the cell wall. Annals of Botany 85, 681–686.

[CIT0069] Shabala S Newman IA Morris J 1997 Oscillations in H^+^ and Ca^2+^ ion fluxes around the elongation region of corn roots and effects of external pH. Plant Physiology 113, 111–118. 12223594 10.1104/pp.113.1.111 PMC158121

[CIT0070] Shabala S Pottosin I 2014 Regulation of potassium transport in plants under hostile conditions: implications for abiotic and biotic stress tolerance. Physiologia Plantarum 151, 257–279. 24506225 10.1111/ppl.12165

[CIT0071] Shabala S White RG Djordjevic MA Ruan YL Mathesius U 2016 *b* Root to shoot signalling: integration of diverse molecules, pathways and functions. Functional Plant Biology 43, 87–104. 10.1071/FP15252 32480444

[CIT0072] Shabala S Wu H Bose J 2015 Salt stress sensing and early signalling events in plant roots: current knowledge and hypothesis. Plant Science 241, 109–119. 26706063 10.1016/j.plantsci.2015.10.003

[CIT0073] Shi H Quintero FJ Pardo JM Zhu JK 2002 The putative plasma membrane Na^+^/H^+^ antiporter SOS1 controls long-distance Na^+^ transport in plants. The Plant Cell 14, 465–477. 11884687 10.1105/tpc.010371 PMC152925

[CIT0074] Smethurst CF Rix K Garnett T Auricht G Bayart A Lane P Wilson SJ Shabala S 2008 Multiple traits associated with salt tolerance in lucerne: revealing the underlying cellular mechanisms. Functional Plant Biology 35, 640–650. 10.1071/FP08030 32688819

[CIT0075] Sun J Chen S Dai S 2009 NaCl-induced alternations of cellular and tissue ion fluxes in roots of salt-resistant and salt-sensitive poplar species. Plant Physiology 149, 1141–1153. 19028881 10.1104/pp.108.129494 PMC2633858

[CIT0076] Sze H Li X Palmgren MG 1999 Energization of plant cell membranes by H^+^-pumping ATPases: regulation and biosynthesis. The Plant Cell 11, 677–690. 10213786 10.1105/tpc.11.4.677 PMC144215

[CIT0077] Véry AA Nieves-Cordones M Daly M Khan I Fizames C Sentenac H 2014 Molecular biology of K^+^ transport across the plant cell membrane: what do we learn from comparison between plant species? Journal of Plant Physiology 171, 748–769. 24666983 10.1016/j.jplph.2014.01.011

[CIT0078] Wu H Zhu M Shabala L Zhou M Shabala S 2015 K^+^ retention in leaf mesophyll, an overlooked component of salinity tolerance mechanism: a case study for barley. Journal of Integrative Plant Biology 57, 171–185. 25040138 10.1111/jipb.12238

[CIT0079] Xie YJ Xu S Han B Wu MZ Yuan XX Han Y Gu Q Xu DK Yang Q Shen WB 2011 Evidence of Arabidopsis salt acclimation induced by up-regulation of HY1 and the regulatory role of RbohD-derived reactive oxygen species synthesis. The Plant Journal 66, 280–292. 21205037 10.1111/j.1365-313X.2011.04488.x

[CIT0080] Yang Y Xu S An L Chen N 2007 NADPH oxidase-dependent hydrogen peroxide production, induced by salinity stress, may be involved in the regulation of total calcium in roots of wheat. Journal of Plant Physiology 164, 1429–1435. 17223222 10.1016/j.jplph.2006.08.009

[CIT0081] Zepeda-Jazo I Velarde-Buendía AM Enríquez-Figueroa R Bose J Shabala S Muñiz-Murguía J Pottosin II 2011 Polyamines interact with hydroxyl radicals in activating Ca^2+^ and K^+^ transport across the root epidermal plasma membranes. Plant Physiology 157, 2167–2180. 21980172 10.1104/pp.111.179671 PMC3327209

[CIT0082] Zhu JK 2001 Plant salt tolerance. Trends in Plant Science 6, 66–71. 11173290 10.1016/s1360-1385(00)01838-0

[CIT0083] Zhu JK 2003 Regulation of ion homeostasis under salt stress. Current Opinion in Plant Biology 6, 441–445. 12972044 10.1016/s1369-5266(03)00085-2

