# Supplementary material for: Difference in root K+ retention ability and reduced sensitivity of K+-permeable channels to reactive oxygen species confer differential salt tolerance in three Brassica species
Source: J Exp Bot. 2016 Jun 23;67(15):4611–25. doi: 10.1093/jxb/erw236 (PMC4973732; doi:10.1093/jxb/erw236)
Supplement: Supplementary Data [file supp_67_15_4611__index.html]

Difference in root K+ retention ability and reduced sensitivity of K+-permeable channels to reactive oxygen species confer differential salt tolerance in three Brassica species — Difference in root K+ retention ability and reduced sensitivity of K+-permeable channels to reactive oxygen species confer differential salt tolerance in three Brassica species — Supplementary Data 

# Difference in root K+ retention ability and reduced sensitivity of K+-permeable channels to reactive oxygen species confer differential salt tolerance in three *Brassica* species

## Supplementary Data

Data files

- supplementary\_Tables\_S1\_S3.pdf - Supplementary Data
