# Supplementary material for: Difference in root K+ retention ability and reduced sensitivity of K+-permeable channels to reactive oxygen species confer differential salt tolerance in three Brassica species
Source: J Exp Bot. 2016 Jun 23;67(15):4611–25. doi: 10.1093/jxb/erw236 (PMC4973732; doi:10.1093/jxb/erw236)
Supplement: Supplementary Data [file supp_erw236_supplementary_Tables_S1_S3.pdf]

Difference in root K^+ retention ability and reduced sensitivity of K^+-permeable channels to ROS confer differential salt tolerance in Brassica.

Koushik Chakraborty, Jay Bose, Lana Shabala, and Sergey Shabala

SUPPLEMENTARY DATA

Supplementary Table S1: List of liquid ionophores and respective back-filling solution used for micro-electrode preparation

Ion	Liquid ionophore (LIX)	Back-filling solution
Na^+	Sodium ionophore Sigma Cat. 129880	500 mM NaCl
Ca^{2+}	Calcium ionophore I-Cocktail Sigma Cat. 21048	500 mM $CaCl_2$
K^+	Potassium ionophore I-Cocktail Sigma Cat. 99311	500 mM KCl
H^+	Hydrogen ionophore II-Cocktail Sigma Cat. 95297	15 mM NaCl, 40 mM KH_2PO_4 (pH 6.0 adjusted with NaOH)

Supplementary Table S2: Nucleotide sequences of different primers used in the study along with amplicon length

Primer	Forward Primer Sequence (5'-3')	Reverse Primer Sequence (5'-3')	Amplicon Size	Reference Gene Bank Accession
BnSOS1	CAAAGGAGGAAGTACAGAGATGG	CAACTGTAGGCCAGTCAGCA	123	EU487184.1
BrAHA1	CAAAGGAGGAAGTACAGAGATGG	GATAGCCATAGTCACGGACAAG	102	GR723037.1
BoBCA1	TCGTCCTCGGCATTCTCAAC	ACCTGGCAAAAGACGAAGGA	112	X99972.1
BnGORK	ACAGATGGAACAGAAGACCTTG	CTCGAGGATGTTGGAGAAAGAC	159	XM_013852302.1<br>NM_123109.4
BnHKT1	ACTCGGTGGTTCTTGGTTATC	AAGTGGAGACCGTTGTGAAG	153	XM_013819627.1
BnAKT1	GGAACATGGTGCTACCATAGAG	CTTGCTCGAGGAGATACTTAACC	200	XM_013888480.1
BnHAK5	CCAAGAGCCAGAAGATAGAGAAC	CCATGGAGGTGCCCATAATAG	152	XM_013850225.1<br>NM_117416.2
Br18s	GGCCGTTCTTAGTTGGTGGA	GCCTAAACGGCCATAGTCCC	130	LC009536.1

Supplementary Table S3: Correlation matrix (two-tailed Pearson's correlation) for different physiological and ion uptake parameters with transcript abundance of key transporters/proteins associated with salt-tolerance in three *Brassica* species

	Root Length	Cell Viability	Steady-state Na^+ flux	Peak Na^+ uptake	Peak K^+-Efflux	Total K^+ leakage	MP	*SOS1*	*AHA1*	*BCA1*	*GORK*	*AKT1*	*HKT1*	*HAK5*
Root Length	1.000	0.958**	-0.818**	-0.892**	-0.892**	-0.878**	0.768*	0.770*	0.773*	0.729*	-0.857**	0.925**	0.627	0.809**
Cell Viability		1.000	-0.857**	-0.927**	-0.931**	-0.919**	0.829**	0.819**	0.812**	0.775*	-0.900**	0.971**	0.670*	0.856**
Steady-state Na^+-flux			1.000	0.961**	0.973**	0.968**	-0.976**	-0.954**	-0.951**	-0.936**	0.972**	-0.944**	-0.894**	-0.959**
Peak Na^+-uptake				1.000	0.997**	0.997**	-0.931**	-0.970**	-0.967**	-0.950**	0.996**	-0.984**	-0.893**	-0.984**
Peak K^+-efflux					1.000	0.994**	-0.947**	-0.968**	-0.963**	-0.946**	0.994**	-0.989**	-0.888**	-0.981**
Total K^+ leakage						1.000	-0.935**	-0.967**	-0.963**	-0.948**	0.998**	-0.977**	-0.891**	-0.981**
MP							1.000	0.943**	0.938**	0.929**	-0.944**	0.929**	0.892**	0.947**
SOS1								1.000	0.998**	0.997**	-0.979**	0.929**	0.974**	0.997**
AHA1									1.000	0.996**	-0.976**	0.923**	0.976**	0.993**
BCA1										1.000	-0.964**	0.899**	0.988**	0.989**
GORK											1.000	-0.972**	-0.916**	-0.989**
AKT1												1.000	0.823**	0.951**
HKT1													1.000	0.955**
HAK5														1.000

** Correlation is significant at the 0.01 level (2-tailed)
* Correlation is significant at the 0.05 level (2-tailed)
